# Bioactive Compounds from Norway Spruce Bark: Comparison Among Sustainable Extraction Techniques for Potential Food Applications

**DOI:** 10.3390/foods8110524

**Published:** 2019-10-23

**Authors:** Sara Spinelli, Cristina Costa, Amalia Conte, Nicola La Porta, Lucia Padalino, Matteo Alessandro Del Nobile

**Affiliations:** 1 Department of Agricultural Sciences, Food and Environment, University of Foggia, Via Napoli, 25–71121 Foggia, Italy; sara.spinelli@unifg.it (S.S.); cristina.costa@unifg.it (C.C.); lucia.padalino@unifg.it (L.P.); matteo.delnobile@unifg.it (M.A.D.N.); 2 Department of Sustainable Agro-ecosystems and Bioresources, Research and Innovation Centre, Fondazione Edmund Mach, Via E. Mach 1, 38010 San Michele all’Adige, Italy; nicola.laporta@fmach.it; 3 The EFI Project Centre on Mountain Forests (MOUNTFOR), Via E. Mach 1, 38010 San Michele all’Adige, Italy

**Keywords:** supercritical fluid extraction, pressurized liquid extraction, ultrasound-assisted extraction, *trans*-resveratrol, Norway spruce bark

## Abstract

*Picea abies* (L.) Karst, (Norway spruce) bark, generally considered as wood industry waste, could potentially be used as a valuable source of antioxidants for food applications. In this study, supercritical fluid extraction (SFE), pressurized liquid extraction (PLE), and ultrasound-assisted extraction (UAE) were carried out in order to recover bioactive compounds from bark of Norway spruce. Obtained results show that PLE with ethanol as solvent was the most effective method for extracting total flavonoid compounds (21.14 ± 1.42 mg quercetin g^−1^ sample) and consequently exerted the highest antioxidant activity measured by 2,2′-azino-bis (3-ethylbenzothiazoline-6-sulfonic acid) (257.11 ± 13.31 mg Trolox g^−1^ sample). On the other hand, UAE extract contained the maximum phenolic concentration (54.97 ± 2.00 mg gallic acid g^−1^ sample) and the most interesting antioxidant activity measured by the ferric-reducing antioxidant power (580.25 ± 25.18 µmol FeSO_4_ g^−1^ sample). Additionally, PLE and UAE have demonstrated great efficiency in the extraction of *trans*-resveratrol, quantified by HPLC (0.19 and 0.29 mg *trans*-RSV g^−1^ sample, respectively).

## 1. Introduction

Annually, a considerable amount of bark waste is generated as by-product from the industrial wood transformation. This waste is usually discarded or used for energy, biogas production, or animal feed [1]. Nevertheless, it is known that tree barks contain a wide variety of bioactive compounds [2]. In particular, several authors have found a large amount of phenolic antioxidants in bark of *Picea abies* (Norway spruce), one of the most distributed conifer species in Eurasian forests [3,4,5]. Spruce bark is especially rich in glycosylated monomeric stilbenes (astringin, piceid, and isorhapontin) and their corresponding aglycone forms (piceatannol, resveratrol, and isorhapontigenin) [6]. Among these different kinds of stilbenes, *trans*-resveratrol (trans-3, 5, 4′-trihydroxystilbene; *trans*-RSV) has attracted great attention. It is a natural polyphenolic compound found in a variety of food and also in bark tree [7]. Actually, it is considered a powerful compound capable to improve health and prevent chronic disease in human [8,9]. Recently published studies have shown that resveratrol can also protect against some neurodegenerative diseases, obesity and diabetes [10], high blood pressure [11], as well as cancer [12] and osteoporosis [9]. In addition, it is widely used in cosmetics and dermatology [13]. Even though basic extractions from spruce bark do not represent a novelty in the literature [1,3,14,15], specific comparisons among available techniques are still very few.

Nowadays, there is a great attention to green extraction technologies able to reduce or eliminate the use of hazardous substances and limit the cost of solvent waste disposal [16]. Among them, a prominent position can be occupied by supercritical fluid extraction (SFE), pressurized liquid extraction (PLE), and ultrasound-assisted extraction (UAE), all considered sustainable techniques [17,18,19]. SFE is considered a fast, efficient, and clean method [20]. Carbon dioxide is the most common gas used as supercritical fluid due to its moderate critical temperature and pressure (31.3 °C and 72.9 atm, respectively) [17]. Applications for the extraction of essential oils, tocotrienols, alkaloids, phenolic compounds, carotenoids, and tocopherols from different food matrices were carried out [17,21,22]. PLE is a technique that uses liquid solvents at elevated pressure and temperature to enhance the extraction performance [19]. The PLE system provides protection to oxygen- and light-sensitive compounds and improves the extraction yield, thus also decreasing time and solvent consumption [19,23,24]. UAE is another efficient extraction method, with high reproducibility, which requires low energy and minimum consumption of solvent. Aromas, phenols, antioxidant pigments, and low-molecular-weight compounds have been extracted by this technique [18]. The extraction of antioxidant compounds and in particular of resveratrol from spruce bark could be an efficient way to reuse and enhance this voluminous biomass waste.

The aim of this work was to compare the extracts of Norway spruce bark obtained by SFE, PLE, and UAE. In particular, total extract yield (TEY), total phenolic content (TPC), total flavonoid content (TFC), and antioxidant capacity were measured to compare the efficacy of each technique. Chromatographic identification of *trans*-RSV was also performed for the extracts with the highest polyphenols content.

## 2. Materials and Methods

### 2.1. Wood Materials and Chemicals

Norway spruce bark was supplied by the timber sawmill company Vender Legnami s.r.l. (Trento, Italy) and dried at room temperature for two weeks. The bark was collected in July 2018, obtained by a stock of timber logs processed and debarked by the company. The spruce logs were coming from Trentino forests (Italy) at their final cutting phase. The trees were growing at an elevation ranging from 1000 to 1600 m a.s.l. The tree age was ranging from 90 to 110 years old. The bark thickness was ranging from 4 to 10 mm. The barks were ground in a knife mill at room temperature, and the powdered bark was sieved to select particles smaller than 1 mm.

Standard *trans*-resveratrol (3,5,4′-trihydroxystilbene) and all analytical grade reagents were purchased from Sigma-Aldrich (Milano, Italy). CO_2_ with purity degree of 4.5 was supplied by Sapio (Monza, Italy), while N_2_ with purity degree of 99.9% was provided by Air Liquide (Milan, Italy).

### 2.2. Supercritical Fluid Extraction

Supercritical fluid extractions from bark were carried out in triplicate with a Speed SFE-2 extractor (Applied Separation, Allentown, PA, USA). In particular, different concentrations of ethanol (10, 20, 40, and 70%; *v*/*v*) were tested (SFE_10, SFE_20, SFE_40, and SFE_70, respectively). According to Talmaciu et al. [14], for aqueous ethanol as co-solvent, a pressure of 100 bar and a temperature of 40 °C for a static time of 150 min and a dynamic time of 105 min were used. An amount of 2 g of spruce bark was used for all the experiments. Extractions were performed with 6 mL/min flow rate in the static phase and with CO_2_ and ethanol as co-solvent, with 10:1 mL/min flow rate ratio for the dynamic phase.

### 2.3. Pressurized Liquid Extraction

Pressurized liquid extractions were performed in triplicate on a PLE-1 system (LabService Analytica srl, Anzola Emilia, Italy) using distillate water at 160 °C (PLE_H_2_O) and absolute ethanol at 180 °C (PLE_EtOH) as solvent, according to Co et al. [3]. In particular, the extraction method included different steps: sample load into cell (30 g); cell preparation (3 min); pressurization and heating (5 min, 50 bar, and 160 °C or 180 °C for ethanol and water, respectively); depressurization (0.1 min); flush volume (60%), and finally, N_2_ purge (2 min). To remove any process carryover, a washing cycle was made among the extractions.

### 2.4. Ultrasound-Assisted Extraction

Ultrasound-assisted extractions were performed in triplicate using an ultrasonic bath CP104 (C.E.I.A., Viciomaggio, Arezzo, Italy; bath frequency 39 kHz, power 200 W). The extraction conditions have been set according to the results obtained from Ghitescu et al. [15] for polyphenol recovery in spruce wood bark. In particular, a process time of 60 min, an extraction temperature of 54 °C, a concentration of ethanol of 70% (*v*/*v*), and a material/solvent ratio of 1:10 were taken into account.

### 2.5. Chemical Characterization

#### 2.5.1. Total Extraction Yield (TEY)

The extracts were evaporated overnight in a vacuum oven (OPTO-LAB, Concordia, Modena, Italy) at 30 °C, and the obtained final mass was weighted to calculate the TEY. Prior to analysis, each extract recovered with ethanol (20 mL) was stored in the dark at 4 °C. Results were expressed as mg of dry extract per gram of samples.

#### 2.5.2. Total Phenolic Content (TPC)

TPC was spectrophotometrically measured using Folin-Ciocalteu reagent, according to conditions previously described by Spinelli et al. [22]. The total phenol contents were evaluated using a standard curve with different gallic acid concentrations (3.125–100 mg L^−1^; *R*^2^ = 0.99). Results were expressed as mg gallic acid equivalents per gram of dry weight (dw).

#### 2.5.3. Total Flavonoid Content (TFC)

The aluminum trichloride method was carried out to determine TFC, as described by Spinelli et al. [22]. The calibration curve was made with standard solutions of quercetin (6.25–400 mg L^−1^; *R*^2^ = 0.99) in order to express the total flavonoid content as mg quercetin equivalent per gram of dry weight (dw).

#### 2.5.4. Antioxidant Activity

The ABTS [2,2′-azino-bis(3-ethylbenzothiazoline-6-sulfonic acid)] assay was measured as described by Marinelli et al. [25]. The ABTS values were calculated from a standard curve of different concentrations of Trolox (3.125–600 mg L^−1^; *R*^2^ = 0.99). The radical scavenging capacity of extracts was quantified as mg Trolox equivalent per gram of dry weight (dw).

The antioxidant capacity of extracts was also estimated in another assay, according to the FRAP (ferric reducing antioxidant power) procedure described by Lucera et al. [26]. For determination, a calibration curve of ferrous sulfate heptahydrate (FeSO_4_·7H_2_O) was prepared, with dilutions from 600 µmol to 12.5 µmol (*R*^2^ = 0.99).

### 2.6. HPLC Analysis of Trans-Resveratrol

Chromatographic identification of *trans*-RSV was performed using an Agilent 1100-Series HPLC system (Agilent Technologies Inc, Santa Clara, CA, USA), equipped with a degasser, binary pump solvent delivery, auto sampler, column oven, and DAD detector. An Agilent Zorbax Eclipse C18 (4.6 × 150 mm; 5 µm particles) and a guard column of the same stationary phase were used for trans-RSV separation. HPLC analysis was performed using the conditions described by Sun et al. [27] with slight modification, consisting of an isocratic elution by methanol–water (40:60 by volume). The flow rate was 1 mL min^−1^, UV detection wavelength 300 nm, injection volume 10 μL, and column temperature 25 °C. The working standard solutions of *trans*-RSV (0.01–100 mg L^−1^) were prepared by diluting the stock solution (250 mg L^−1^) in mobile phase and stored at 4 °C in darkness to avoid oxidative degradation and isomerization of *trans*-RSV to *cis*-form. The method linearity was up to 100 mg L^−1^. The identification of *trans*-RSV in the extracts was performed by comparison of the retention time (∼7.5 min) and UV spectra with *trans*-RSV standard.

### 2.7. Statistical Analysis

A one-way ANOVA and a post-hoc Fisher’s test were used to evaluate statistically significant differences among samples. The software was Statistica 7.1 for Windows (StatSoft Inc., Tulsa, OK, USA). All tests were carried out in triplicate.

## 3. Results and Discussion

In this study, three different extraction techniques, that is, supercritical fluid extraction, pressurized liquid extraction, and ultrasound-assisted extraction, were compared in order to obtain a valuable spruce bark extract rich in bioactive compounds with high antioxidant activity.

### 3.1. Supercritical Fluid Extraction

Table 1 summarizes the experimental results of the SFE, in terms of total extraction yield (TEY), total phenolic content (TFC), total flavonoid content (TFC), and antioxidant activity.

Concerning the TEY, the amount of ethanol did not influence the extraction yield. Comparable results were recorded, even though some differences in the chemical composition of the extract were found. As can be seen in the Table 1, the gradual increase of ethanol in the SFE statistically improves the extraction capacity of phenolic compounds, flavonoids, and antioxidant activity of spruce extracts [28]. In particular, TPC steadily increased as the ethanol concentration increased (from 0.77 ± 0.02 to 2.50 ± 0.03 mg GAEs/g dw), and a similar behavior was also observed for TFC, with the highest value 1.75 ± 0.10 mg QEs/g dw in the assay SFE_40. Similarly, SFE_40 shows the radical scavenging ABTS and FRAP capacity respectively 2 and 3 times higher than that obtained with SFE_10. It is well known that ethanol promotes the recovery of polar compounds from the samples due to changes in the extractive properties (diffusivity, density, and viscosity); in fact, the purpose of ethanol is to swell plant cells, thus allowing both solvent penetration and diffusion of the solute in the solid matrix. In this way, both the increase in polarity of supercritical CO_2_ and the rapid formation of interactions with the analyte of interest are promoted [29]. A similar behavior was also reported in several previous studies that demonstrated how ethanol enhanced the extraction of bioactive compounds and consequently improved the antioxidant activity. Conde et al. [30] used supercritical CO_2_ to extract phenolic compounds from *Pinus pinaster* wood and noted that the extraction yield and the phenolic concentration increased when ethanol was used as co-solvent. Fabrowska et al. [31] also developed a supercritical fluid extraction process in order to revalorize different freshwater green macro-algae species, demonstrating that the increase in the concentration of ethanol from 0% to 15% resulted in an increase in the extraction yield and in bioactive compound concentrations. Our assay with 70% of ethanol solution allowed recording extract statistically poorer in phenol and flavonoid content compared with the assay with lower ethanol concentrations (data not shown). As also reported in the literature, the use of high concentration of co-solvent can sometimes provoke reduction of target bioactive compounds, due to interactions between CO_2_ and co-solvent [32].

### 3.2. Pressurized Liquid and Ultrasound-Assisted Extraction

In Table 2 are reported the TEY, TPC, TFC, and the antioxidant activity for Norway spruce bark extracts obtained by PLE and UAE techniques.

As regards PLE extract, from a general point of view, the technique is more efficient than SFE, because the working conditions of PLE generally allow protecting of bioactive compounds [33]. In particular, Rostagno et al. [34] described how isoflavones can be extracted by PLE from soybeans without degradation. As can be observed in Table 2, the two different tested solvents did not influence significantly the yield. On the contrary, higher content in TPC, TFC, and radical scavenging ABTS and FRAP were obtained for PLE extraction with absolute ethanol compared with water. Howard and Pandjaitan [35] reported that the flavonoids extracted from spinach by PLE with ethanol were more effective when compared with the same compounds extracted by different conditions. The significant increase in terms of antioxidant capacity, measured by ABTS or FRAP is also in accordance with literature data. Zhao et al. [36] also reported high DPPH (2,2-diphenyl-1-picryl-hydrazyl-hydrate) radical scavenging capacity and total phenolic content in barley extract with ethanol, compared with water extract. In the PLE technique, the application of high temperature during the extraction significantly decreases the dielectric constant of water and cuts down the surface tension, thus promoting the extraction of bioactive compounds, but PLE water extraction can damage some thermolabile compounds, such as polyphenols and flavonoids, thus justifying the preference for ethanol as co-solvent [23].

Results obtained from UAE extraction highlighted that UAE yield was higher than SFE_40 yield and comparable to that of the PLE_EtOH extract. As can be seen in the Table 2, UAE extract exerted the maximum phenolic concentration among the various green techniques adopted (54.97 ± 2.00 mg GAEs/g dw). The literature also confirms that UAE applied to various natural matrices significantly increases the phenolic compounds extracted, compared with alternative extraction methods [19,23]. As a fact, the production of cavitation bubbles promotes better extraction yield and increases the antioxidant activity of these extracts [37]. A similar trend was also observed when the comparison was made in terms of FRAP-antioxidant activity.

In order to highlight some possible relationships among the values reported in Table 2, a remarkable correlation between TPC and FRAP assay can be found with both the extraction methods adopted [22]. Thaipong et al. [38], studying the comparison among ABTS, DPPH, FRAP, and ORAC assays for estimating the antioxidant activity of guava fruit extracts, also demonstrated that FRAP test showed high correlation with total phenolic content. Youn et al. [39] also investigated the relationship between antioxidant activity and polyphenol or flavonoid contents in leaf extracts obtained from *Dendropanax morbifera* LEV. and showed that FRAP value was strongly correlated with polyphenols. A similar correlation can be also observed between total flavonoid compounds and ABTS. Similar trends were also found in other literature data carried out on various medicinal plants [40,41].

The different mechanism of action between FRAP and ABTS assay justifies the different values recorded between them. As a fact, the FRAP assay is based on the singlet electron transfer, while ABTS is based on the mixed mode with singlet electron transfer and hydrogen atom transfer [42].

### 3.3. Chromatographic Identification of Trans-RSV

**Figure 1:**
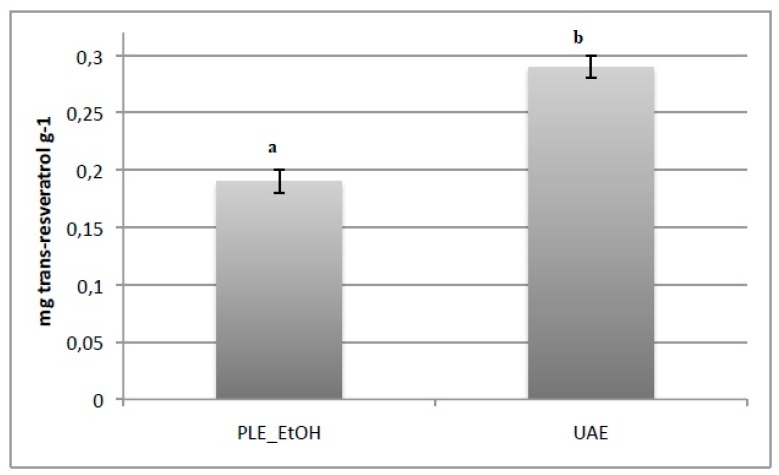
*Trans*-resveratrol content in PLE and UAE extracts. Samples with different superscript letters differ significantly (*p* < 0.05).

The spruce bark extracts obtained by the two best extraction techniques in terms of total phenol content (PLE_EtOH and UAE) were also analyzed by chromatographic identification in order to achieve a quantitative and complete characterization of *trans*-RSV. In Figure 1 is reported the *trans*-RSV content in PLE_EtOH and UAE extracts. As can be observed, higher levels of *trans*-RSV were found in UAE extract (0.29 mg/g dw). Extraction conditions and isomerization to *cis* isomer can be the explanation to justify the low content of *trans*-RSV in PLE extract [43]. Zupancic et al. [44] also highlight that pH, temperature, and different extraction methods influenced *trans*-RSV stability. Garcìa-Pèrez et al. [45] found resveratrol as the only stilbenes in *Picea marina* bark extract with ethyl acetate. Differently, Co et al. [3] identified resveratrol in spruce extract with PLE by nuclear magnetic resonance and mass spectrometry detection.

## 4. Conclusions

The results presented in this research show valid means to obtain antioxidant compounds, with a better focus on resveratrol, from Norway spruce bark using different environmental-friendly extraction techniques. In particular, the best results in terms of total phenolic compounds and antioxidant capacity measured by FRAP assays were obtained for UAE extract. PLE extract obtained with absolute ethanol shows the highest total flavonoid content and the best antioxidant capacity measured by ABTS assays. The highest *trans*-RSV content identified by chromatography was recorded for the UAE extract (0.29 mg/g dw), thus suggesting the potential of ultrasound-assisted extraction with ethanol (70% *v*/*v*) for the valorization of waste, to record antioxidant compounds that can be applied to food and pharmaceutical sectors.

## Figures and Tables

**Table 1:** Total extraction yield (TEY), total phenolic content (TPC), total flavonoid content (TFC), and antioxidant activity (ABTS and FRAP) of SFE spruce extracts with different ethanol concentrations: SFE_10 (10%; *v*/*v*); SFE_20 (20%; *v*/*v*); SFE_40 (40%; *v*/*v*).

	TEY	TPC	TFC	ABTS	FRAP
	mg/g dw	mg GAEs/g dw	mg QEs/g dw	mg TEs/g dw	µmol FeSO_4_·7H_2_O/g dw
SFE_10	28.6 ± 0.36 ^a^	0.77 ± 0.02 ^a^	0.47 ± 0.02 ^a^	2.48 ± 0.13 ^a^	8.31 ± 0.24 ^a^
SFE_20	30.7 ± 0.96 ^a^	1.24 ± 0.07 ^b^	1.05 ± 0.15 ^b^	3.08 ± 0.16 ^b^	10.01 ± 0.81 ^b^
SFE_40	31.2 ± 0.21 ^a^	2.50 ± 0.03 ^c^	1.75 ± 0.10 ^c^	5.29 ± 0.04 ^c^	25.49 ± 0.66 ^c^

Values are means of three replications ± standard deviation. Values in the same column followed by different superscript letters differ significantly (*p* < 0.05). SFE: supercritical fluid extraction. GAEs: gallic acid equivalents; QEs: quercetin equivalent; TEs: Trolox equivalent; FeSO_4_·7H_2_O: ferrous sulfate heptahydrate; ABTS: 2,2′-azinobis (3-ethylbenzothiazoline-6-sulfonic acid). FRAP: ferric reducing antioxidant power.

**Table 2:** Total extraction yield (TEY), total phenolic content (TPC), total flavonoid content (TFC), and antioxidant activity (ABTS and FRAP) of PLE and UAE spruce extracts with water (PLE_H_2_O) and absolute ethanol (PLE_EtOH and UAE_EtOH).

	TEY	TPC	TFC	ABTS	FRAP
	mg/g dw	mg GAEs/g dw	mg QEs/g dw	mg TEs/g dw	µmol FeSO_4_·7H_2_O/g dw
PLE_H_2_O	130.7 ± 8.62 ^a^	33.45 ± 1.44 ^a^	19.03 ± 0.98 ^a^	69.87 ± 1.46 ^c^	389.10 ± 16.87 ^b^
PLE_EtOH	127.9 ± 2.52 ^a^	46.32 ± 2.17 ^b^	21.14 ± 1.42 ^a^	257.11 ± 13.31 ^a^	506.10 ± 31.37 ^a^
UAE_EtOH	123.3 ± 5.77 ^a^	54.97 ± 2.00 ^c^	14.44 ± 1.31 ^b^	128.47 ± 8.61 ^b^	580.25 ± 25.18 ^a^

^a–c^ Values are means of three replications ± standard deviation. Values in the same column followed by different superscript letters differ significantly (*p* < 0.05). PLE: pressurized liquid extraction. UAE: ultrasound-assisted extraction.
